# Hydroxysulfobetaine Surfactant Uptake Regulates the Transport Behavior of Sulfonated Polyacrylamide Soft Microgels for Deep Profile Control

**DOI:** 10.3390/gels12050445

**Published:** 2026-05-19

**Authors:** Jianbing Li, Liwei Niu

**Affiliations:** School of Petroleum Engineering, Hebei Petroleum University of Technology, Chengde 067000, China; lijianbing@vip.sina.com

**Keywords:** sulfonated polyacrylamide soft microgels, hydroxysulfobetaine surfactant, surfactant uptake, transport behavior, deep profile control

## Abstract

To improve the effectiveness of sulfonated polyacrylamide soft microgels (SMGs) in deep profile control, this study investigated a surfactant-assisted regulation strategy based on surfactant uptake and surfactant–microgel association. The uptake behavior of a hydroxysulfobetaine surfactant by SMGs was characterized, and the resulting changes in swelling, frequency-dependent elastic response, electrostatic stabilization, shear resistance, and long-distance transport were evaluated. The surfactant uptake process was well described by pseudo-second-order kinetics and a Langmuir-type saturation model, while FTIR and XPS analyses provided spectroscopic evidence for surfactant association with SMGs, especially at the particle surface. Compared with the SMG system, surfactant addition mildly reduced the swollen median size (*D*_50_) at 15 d from 15.72 to 14.90 μm, and the corresponding swelling ratio decreased slightly but remained above 6.45. The S/SMG system also showed a larger magnitude of negative zeta potential, maintaining a value of −38.5 mV after 60 d compared with −32.1 mV for the SMG system, and generally better shear resistance, with particle size retention 0.8–3.8 percentage points higher over 0–7 d of swelling. Serial core-flooding experiments showed improved deep transport behavior. Although the SMG system produced slightly higher injection pressure below 2.4 m, the S/SMG system maintained a slightly higher pressure response beyond this distance. These results demonstrate that surfactant uptake and surface/network association regulate SMG physicochemical properties, thereby improving their transport and deep profile-control performance.

## 1. Introduction

With the continuous growth in global energy demand and the progressive depletion of conventional oil resources, enhanced oil recovery (EOR) technologies have become increasingly important for exploiting the remaining potential of mature oilfields and improving ultimate oil recovery [1,2,3,4]. Among various EOR methods, chemical flooding plays an important role because it can selectively modify the interactions among injected fluids, reservoir rocks, and resident fluids [5,6,7]. Conventional chemical flooding mainly uses polymers to improve mobility control and sweep efficiency, and surfactants to reduce oil–water interfacial tension and improve microscopic displacement efficiency [8]. However, single-agent chemical systems often show substantial limitations in heterogeneous reservoirs. Polymer flooding can suppress water channeling through high-permeability pathways to some extent, but its profile-control capability is limited in strongly heterogeneous formations or reservoirs containing large pore channels. In addition, polymer molecules are susceptible to mechanical shear, thermal degradation, and salinity fluctuations [9]. Surfactant flooding is also constrained by adsorption loss, chromatographic separation, and inefficient delivery through low-permeability pore throats [10,11]. Therefore, when both sweep efficiency and microscopic displacement efficiency need to be improved simultaneously, single-agent systems often fail to provide balanced performance, resulting in limited overall improvement in oil recovery [12,13,14,15,16].

In this context, surfactant–soft microgel composite systems have attracted increasing attention because they combine the interfacial activity of surfactants with the deep profile-control capability of soft microgels in a single formulation [17,18,19,20,21]. Sulfonated polyacrylamide soft microgels (SMGs) used for profile control are distinct from linear polymers. They are preformed, crosslinked, swellable polymer particles that exhibit gel-like deformability after hydration in aqueous media. Accordingly, their function does not rely primarily on bulk viscosity for mobility control, but is governed mainly by transport, retention, deformation, and dynamic resistance development in porous media [14,22,23,24,25]. These characteristics give SMGs clear potential for in-depth conformance control and flow diversion. Meanwhile, surfactant addition may further improve oil recovery not only by reducing oil–water interfacial tension and promoting emulsification, but also by altering the physicochemical behavior of the microgel system. Many existing studies on surfactant–microgel composite systems have therefore focused on interfacial tension, concentration optimization, particle size, swelling behavior, zeta potential, dispersion stability, and oil-displacement performance [19,20,21,26,27,28].

These studies provide useful guidance for formulation design. However, most of them have focused either on the contribution of surfactants to interfacial properties or on the role of microgels in the overall displacement system. Much less attention has been paid to the reciprocal effect, namely, how surfactant addition changes microgel properties and thereby regulates microgel transport behavior in porous media. This issue is important because the effectiveness of SMGs in deep profile control depends strongly on whether they can maintain mobility and structural integrity during long-distance migration without excessive aggregation, premature plugging, or shear-induced damage [29,30,31]. In particular, it remains unclear how surfactant–microgel interactions are translated into pressure evolution during migration and, ultimately, into deep profile-control performance.

In addition to reducing oil–water interfacial tension and promoting emulsification, surfactant molecules may associate with SMGs through adsorption at the outer interface as well as penetration or partitioning into the hydrated microgel network. Such surfactant uptake and surface/network association can modify swelling behavior, surface charge, dispersion stability, deformation capability, and resistance to shear-induced damage [26,27,28,32]. These changes may in turn determine whether SMGs can pass through pore throats, avoid premature accumulation in the near-wellbore region, and establish effective flow resistance in deeper parts of the reservoir. Therefore, clarifying the link between surfactant uptake, transport-related microgel properties, and migration performance is necessary for the targeted design of surfactant–soft microgel composite systems.

Accordingly, this study investigates how a hydroxysulfobetaine surfactant regulates the transport behavior of SMGs and its implications for deep profile control. The uptake behavior of the surfactant by SMGs was first analyzed to clarify surfactant–microgel association. The resulting changes in swelling, frequency-dependent elastic response, zeta potential, dispersion stability, and shear resistance were then systematically characterized. Finally, long-distance transport tests and serial core-flooding experiments were conducted to evaluate how these coupled effects influenced pressure evolution and deep profile-control performance.

## 2. Results and Discussion

### 2.1. Uptake and Association Behavior of Surfactant with Soft Microgels (SMGs)

#### 2.1.1. Surfactant Uptake Kinetics and Equilibrium Characteristics

**Figure 1:**
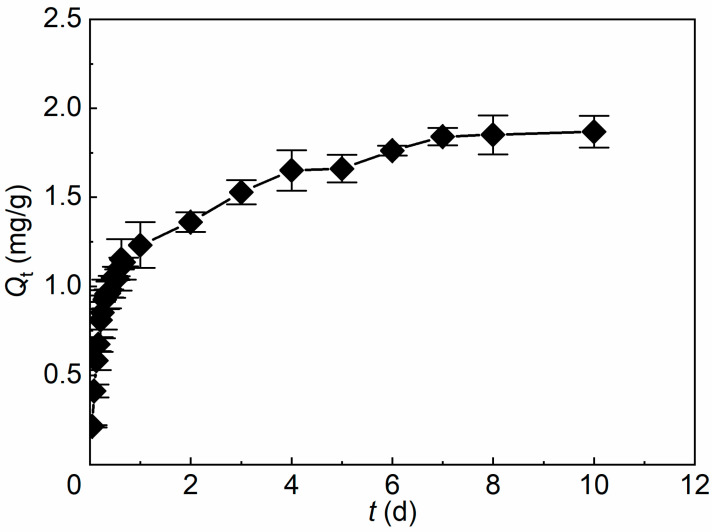
Uptake kinetics of the hydroxysulfobetaine surfactant by soft microgels (SMGs). Symbols and error bars represent mean ± SD (*n* = 3); the line is used only as a guide to the eye.

The evolution of surfactant uptake by soft microgels (SMGs) with contact time is shown in Figure 1. The apparent uptake capacity increased rapidly at the initial stage and then gradually approached equilibrium. Based on the experimental trend, the uptake process can be divided into three stages.

In Stage I (0–1 d), surfactant uptake increased rapidly. The mean *Q*_t_ increased from 0.215 ± 0.006 mg/g at 0.04 d to 1.231 ± 0.129 mg/g at 1 d, reaching approximately 66% of the apparent equilibrium uptake capacity. This result indicates that surfactant molecules could quickly associate with accessible regions of SMGs, including the outer interface and near-surface hydrated network.

In Stage II (1–7 d), the uptake rate decreased markedly, whereas the mean *Q*_t_ continued to increase gradually from 1.231 ± 0.129 to 1.841 ± 0.049 mg/g. This stage suggests a slower uptake process after the initial rapid association, likely involving further occupation of less accessible regions, penetration or partitioning into the hydrated microgel network, and rearrangement of associated surfactant molecules.

In Stage III (>7 d), the mean *Q*_t_ changed only slightly and fluctuated around 1.85–1.87 mg/g, indicating that surfactant uptake had approached dynamic equilibrium. The experimental apparent equilibrium uptake capacity was therefore taken as approximately 1.868 ± 0.089 mg/g.

To quantitatively describe the uptake kinetics, the mean apparent uptake capacities were fitted using the pseudo-first-order model (Equation (1)) and pseudo-second-order model (Equation (2)) [33,34].

(1) $$ln(Q_{e}-Q_{t})=lnQ_{e}-k_{1}t,$$

(2) $$\frac{t}{Q_{t}}=\frac{1}{k_{2}Q_{e}^{2}}+\frac{t}{Q_{e}},$$

where *Q*_e_ and *Q*_t_ (mg/g) are the apparent equilibrium uptake capacity and the apparent uptake capacity at time *t*, respectively; *k*_1_ (day^−1^) and *k*_2_ (g·mg^−1^·day^−1^) are the pseudo-first-order and pseudo-second-order rate constants.

The fitted parameters are summarized in Table 1. The pseudo-first-order model gave a calculated apparent equilibrium uptake capacity of 1.657 mg/g with an *R*^2^ value of 0.8929, whereas the pseudo-second-order model gave a *Q*_e,calc_ value of 1.803 mg/g with a higher *R*^2^ value of 0.9650. In addition, the *Q*_e,calc_ value from the pseudo-second-order model was closer to the experimental equilibrium value (*Q*_e,exp_ = 1.868 ± 0.089 mg/g). These results indicate that the pseudo-second-order model provides a better description of the overall uptake kinetics of the hydroxysulfobetaine surfactant by SMGs.

However, the better fit of the pseudo-second-order model does not by itself demonstrate an exclusively chemisorption-controlled or surface-confined process. Instead, it suggests that the uptake rate is associated with site occupation, surfactant partitioning within the hydrated microgel network, and molecular rearrangement, in addition to mass-transfer effects. Combined with the experimentally observed three-stage behavior, the interaction between the hydroxysulfobetaine surfactant and SMGs can be regarded as a progressive uptake and association process characterized by rapid initial uptake followed by slower equilibration.

#### 2.1.2. Uptake Isotherm and Model Fitting

**Figure 2:**
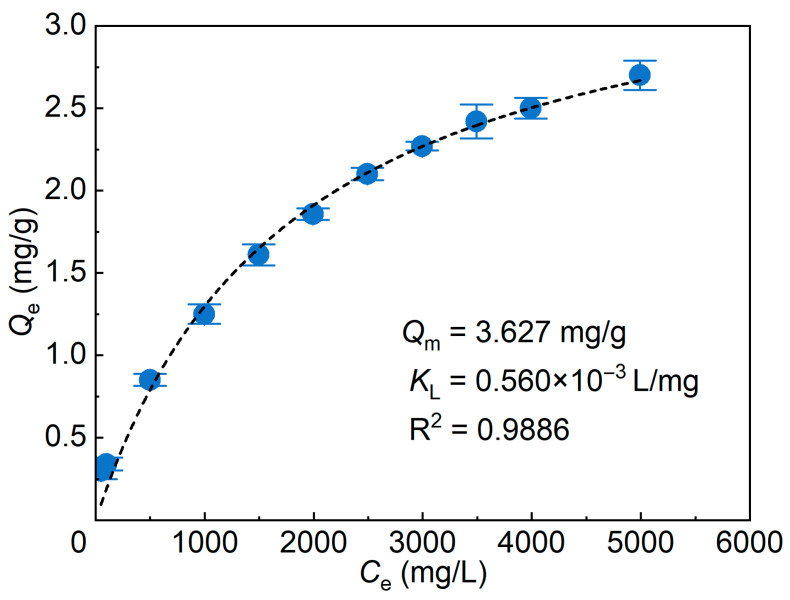
Uptake isotherm of the hydroxysulfobetaine surfactant by SMGs and Langmuir-type model fitting. Symbols and error bars represent mean ± SD (*n* = 3), and the dashed line represents Langmuir fitting based on the mean values.

To characterize the equilibrium uptake behavior of the hydroxysulfobetaine surfactant by SMGs, the apparent equilibrium uptake capacity (*Q*_e_) was measured at different equilibrium concentrations (*C*_e_), as shown in Figure 2. The data are presented as mean ± SD from three independent measurements. The isotherm showed a typical Langmuir-type trend. At relatively low *C*_e_, *Q*_e_ increased rapidly, indicating that many accessible association regions within or near the SMGs were still available. As *C*_e_ increased further, the increment in *Q*_e_ gradually decreased, and the isotherm approached a plateau, indicating progressive saturation of the accessible surfactant-association capacity of the SMGs.

The experimental mean values were fitted using the Langmuir-type saturation model (Equation (3)):

(3) $$\frac{C_{e}}{Q_{e}}=\frac{1}{K_{L}Q_{m}}+\frac{C_{e}}{Q_{m}},$$

where *Q*_m_ (mg/g) is the theoretical maximum apparent uptake capacity and *K*_L_ (L/mg) is the Langmuir-type constant representing the affinity of surfactant association with SMGs.

The Langmuir model described the experimental mean values well, with an *R*^2^ value of 0.9886. The fitted parameters gave a theoretical maximum apparent uptake capacity (*Q*_m_) of 3.627 mg/g and a Langmuir-type constant (*K*_L_) of 0.560 × 10^−3^ L/mg. At the highest tested equilibrium concentration of approximately 4991.9 mg/L, the mean *Q*_e_ reached 2.70 ± 0.089 mg/g, indicating that the apparent uptake capacity continued to increase, but the incremental uptake became smaller at high surfactant concentrations. This plateau-approaching behavior indicates that, once the accessible surfactant-association capacity of the SMGs is close to saturation, further increasing surfactant concentration produces only limited additional uptake.

These results indicate that the interaction between the hydroxysulfobetaine surfactant and SMGs shows a saturable uptake behavior under the tested conditions. This surfactant uptake behavior provides the basis for the subsequent discussion of surfactant-induced changes in transport-related microgel properties.

#### 2.1.3. Spectroscopic Evidence for Surfactant Association with SMGs

FTIR and XPS analyses were performed to provide spectroscopic evidence for the association of the hydroxysulfobetaine surfactant with SMGs. Pristine SMGs, the pure hydroxysulfobetaine surfactant, and washed S/SMG samples were characterized. The washed S/SMG sample was collected after uptake equilibrium, gently washed to remove unbound surfactant, and dried before spectroscopic analysis. Therefore, the surfactant-related signals remaining in washed S/SMG can be attributed to surfactant species associated with SMGs rather than freely retained surfactant in the continuous aqueous phase. Because XPS is surface-sensitive, it mainly reflects surfactant association near the particle surface.

**Figure 3:**
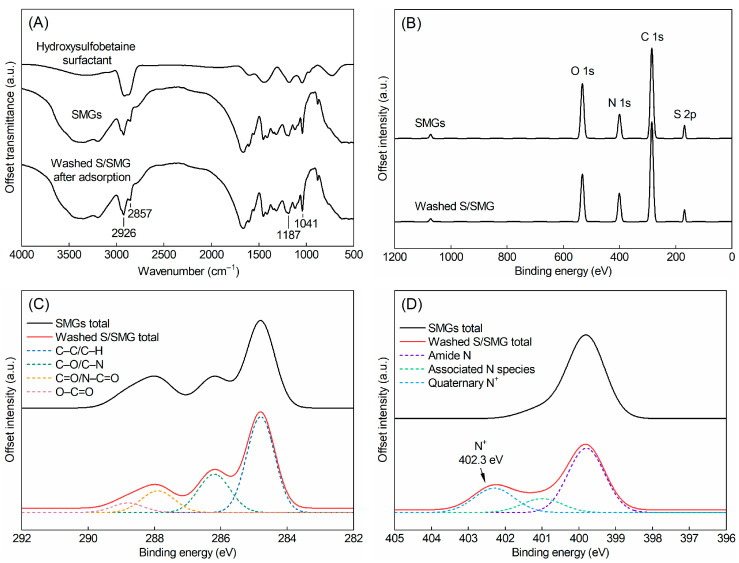
Spectroscopic evidence for hydroxysulfobetaine surfactant association with SMGs: (**A**) FTIR spectra of pristine SMGs, pure hydroxysulfobetaine surfactant, and washed S/SMG after surfactant uptake; (**B**) XPS survey spectra of pristine SMGs and washed S/SMG; (**C**) high-resolution C 1s spectra of pristine SMGs and washed S/SMG; and (**D**) high-resolution N 1s spectra of pristine SMGs and washed S/SMG. The spectra were vertically offset for clarity.

The FTIR spectra are shown in Figure 3A. Pristine SMGs exhibited characteristic absorption bands associated with the polymer network. The broad absorption band at 3350 cm^−1^ and the band at 3198 cm^−1^ can be assigned to O–H/N–H and N–H stretching vibrations, respectively. The peaks at 2926 and 2857 cm^−1^ correspond to the asymmetric and symmetric stretching vibrations of aliphatic C–H groups. The strong band at 1665 cm^−1^ was attributed to the amide I vibration, mainly arising from C=O stretching in amide-containing units. The bands at 1187 and 1041 cm^−1^ can be assigned mainly to SO_3_^−^/S=O stretching vibrations of sulfonic or sulfonate groups, with possible overlap from C–O–C/C–O vibrations.

The pure hydroxysulfobetaine surfactant showed characteristic absorption bands associated with its hydrophobic chain and zwitterionic head group. The strong bands at approximately 2920 and 2850 cm^−1^ were assigned to the asymmetric and symmetric stretching vibrations of methylene groups in the hydrophobic alkyl chain. The absorption bands in the range of 1200–1030 cm^−1^ were mainly associated with overlapping contributions from sulfonate S=O stretching and C–O–C/C–O stretching vibrations from the ether- and hydroxyl-containing segments. After surfactant uptake and washing, washed S/SMG retained the main characteristic bands of SMGs but showed enhanced absorption at approximately 2926 and 2857 cm^−1^ and changes in the 1200–1030 cm^−1^ region. These changes indicate that surfactant-derived alkyl-chain and polar head-group structures remained associated with SMGs after washing. However, because both SMGs and the hydroxysulfobetaine surfactant contain sulfonate groups, the S=O-related FTIR bands alone cannot be used as exclusive evidence for surfactant association.

XPS analysis was further conducted to examine the changes in surface elemental composition and chemical states after surfactant association. As shown in Figure 3B, both pristine SMGs and washed S/SMG exhibited C 1s, O 1s, N 1s, and S 2p signals, which are consistent with the sulfonated and amide-containing structure of SMGs. Because sulfonate groups are present in both SMGs and the hydroxysulfobetaine surfactant, the S 2p signal observed in the survey spectra alone cannot serve as exclusive evidence for surfactant association. Therefore, high-resolution C 1s and N 1s spectra were further analyzed to identify the chemical-state changes associated with surfactant association.

The high-resolution C 1s spectra are shown in Figure 3C. Compared with pristine SMGs, washed S/SMG showed changes in the relative contributions of different carbon-containing species. The C 1s spectrum of washed S/SMG was deconvoluted into C–C/C–H, C–O/C–N, C=O/N–C=O, and O–C=O components. The contribution of the C–C/C–H component is consistent with the presence of alkyl-chain structures from the hydroxysulfobetaine surfactant. Changes in the C–O/C–N component can be related to ether linkages, hydroxyl-containing groups, and C–N species associated with the hydroxysulfobetaine surfactant. These changes indicate that the carbon-containing chemical environment of SMGs, especially near the particle surface, was modified after surfactant association.

The high-resolution N 1s spectra provide more direct surface-sensitive evidence for surfactant association with SMGs (Figure 3D). For pristine SMGs, the N 1s signal was mainly associated with amide nitrogen in the polymer network. After surfactant association, washed S/SMG exhibited an additional high-binding-energy component at 402.3 eV, which can be assigned to quaternary ammonium nitrogen (N^+^) from the hydroxysulfobetaine surfactant. The presence of this N^+^ component after washing demonstrates that the zwitterionic head groups of the surfactant remained associated with the SMG surface. Therefore, the N 1s spectra provide key surface-sensitive evidence for the association of the hydroxysulfobetaine surfactant with SMGs.

Overall, the UPLC–ELSD results quantified the apparent uptake amount of the surfactant by SMGs, whereas the FTIR and XPS results provided spectroscopic evidence for the association of the hydroxysulfobetaine surfactant with SMGs, especially near the particle surface. These results demonstrate that the decrease in residual surfactant concentration was accompanied by surfactant association with SMGs, including association near the particle surface. This surfactant association provides the physicochemical basis for the subsequent regulation of swelling, elastic response, dispersion stability, shear resistance, and transport behavior.

### 2.2. Surfactant-Uptake-Mediated Regulation of SMG Properties Relevant to Transport

#### 2.2.1. Swelling Regulation and Particle Size Evolution

**Figure 4:**
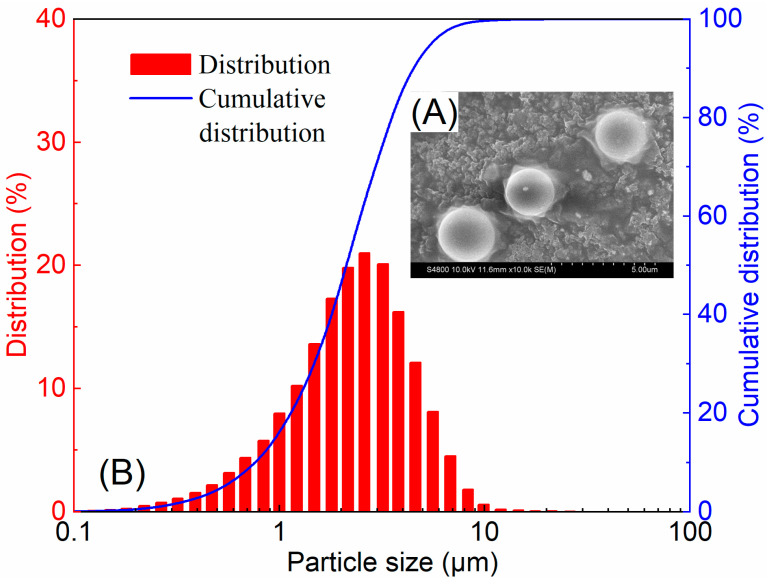
Initial morphology and particle size distribution of soft microgels (SMGs): (**A**) representative SEM image and (**B**) representative particle size distribution and cumulative distribution. The initial median particle size (*D*_50_) was 2.21 ± 0.22 μm, as determined from three independent measurements. Adapted from ref. [35] under the terms of the Creative Commons Attribution 4.0 International License CC BY 4.0).

Figure 4 presents the initial morphology and particle size distribution of the soft microgels (SMGs). The representative SEM image (Figure 4A) shows that the particles were nearly spherical and generally well dispersed. Laser particle size analysis (Figure 4B) gave an initial median diameter (*D*_50_) of 2.21 ± 0.22 μm, which was used as the reference for evaluating swelling-induced size evolution.

**Figure 5:**
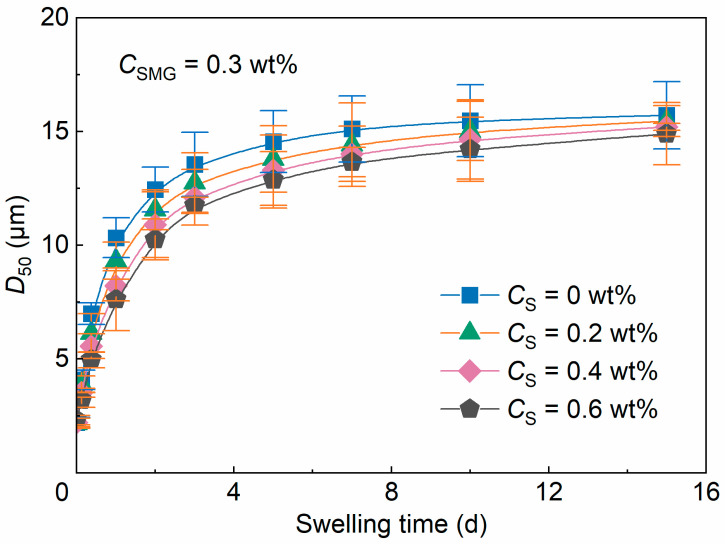
Evolution of median particle size (*D*_50_) with swelling time at different surfactant concentrations (*C*_SMG_ = 0.3 wt%). Symbols and error bars represent mean ± SD (*n* = 3), and the lines are used only as guides to the eye.

The swelling behavior of the SMG system and S/SMG systems is shown in Figure 5. For clarity, only representative surfactant concentrations (*C*_S_ = 0, 0.2, 0.4, and 0.6 wt%) are presented, as the intermediate concentrations showed the same overall trend. All systems exhibited two-stage swelling behavior, with a rapid increase in *D*_50_ during the first 2 d of swelling followed by a slower increase at longer swelling times, approaching a quasi-equilibrium state after 7–10 d.

Surfactant addition produced a moderate concentration-dependent decrease in the mean swollen particle size. As *C*_S_ increased from 0 to 0.6 wt%, the mean *D*_50_ at the same swelling time generally decreased. At 15 d, for example, *D*_50_ decreased from 15.72 ± 1.49 μm for the SMG system to 14.90 ± 1.37 μm for the S/SMG system containing 0.6 wt% surfactant, corresponding to an approximate reduction of 5.2% based on the mean values.

Consistent with the decrease in swollen *D*_50_, the calculated swelling ratio also decreased slightly with surfactant addition. Using the corresponding initial *D*_50_ values as references, the swelling ratio at 15 d decreased from 7.09 for the SMG system to 6.45 for the S/SMG system containing 0.6 wt% surfactant. Therefore, surfactant addition did reduce both the swollen particle size and the corresponding swelling ratio. However, all systems still showed swelling ratios above 6.45, indicating that the microgels retained substantial swelling ability under the tested conditions. This result suggests that surfactant addition limited excessive swelling rather than eliminating the swelling capability of SMGs. This behavior is consistent with surfactant uptake and association with SMGs, as supported by the UPLC–ELSD, FTIR, and XPS results in Section 2.1.3. Surfactant molecules may penetrate or partition into the hydrated microgel network, where they may alter water uptake, polymer-chain interactions, local hydration state, and osmotic balance, thereby moderately reducing the equilibrium swollen size. Such controlled swelling may help limit excessive near-wellbore expansion and favor deeper migration in porous media.

#### 2.2.2. Frequency-Dependent Elastic Response and Deformation Capability

**Figure 6:**
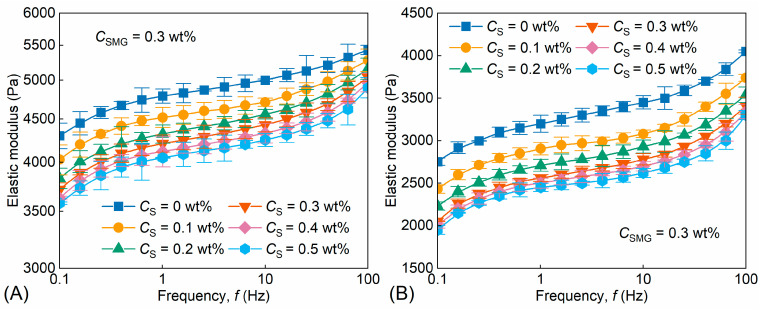
Frequency-dependent elastic modulus of SMG and S/SMG systems at different surfactant concentrations (CS = 0.0–0.5 wt%): (**A**) before swelling and (**B**) after swelling equilibrium (45 °C, 7 d). Symbols and error bars represent mean ± SD (*n* = 3), and the lines are used only as guides to the eye.

The elastic response of soft microgels is closely related to their deformation–recovery behavior and their ability to generate local flow resistance during profile control. Unlike linear polymer solutions, SMGs do not primarily rely on bulk viscosity for mobility control; instead, their transport and plugging behavior are governed mainly by particle swelling, deformation, retention, and elastic recovery. Therefore, the elastic modulus was used here as a deformation-related parameter. Figure 6 shows the frequency-dependent elastic modulus of the SMG system and S/SMG systems at different surfactant concentrations before swelling (Figure 6A) and after swelling equilibrium (Figure 6B, 45 °C, 7 d).

As shown in Figure 6, the elastic modulus increased with oscillation frequency, *f*, over the range of 0.1–100 Hz for all systems, indicating a frequency-dependent elastic response of the packed swollen microgels. The overall frequency dependence remained similar at different surfactant concentrations, suggesting that surfactant addition did not change the qualitative elastic-response pattern of the microgels but mainly affected the modulus magnitude.

Before swelling, surfactant addition produced a concentration-dependent decrease in the mean elastic modulus (Figure 6A). At 0.1 Hz, for example, the elastic modulus decreased from 4300 ± 150 Pa for the SMG system to 3580 ± 12 Pa for the S/SMG system containing 0.5 wt% surfactant, corresponding to an approximate reduction of 16.7% based on the mean values. The difference between adjacent curves became less pronounced above approximately 0.2–0.3 wt% surfactant, indicating a diminishing incremental effect at higher surfactant concentrations. This trend is consistent with the uptake saturation tendency discussed in Section 2.1.2.

After swelling equilibrium, the elastic modulus decreased for all systems (Figure 6B). For the SMG system at 0.1 Hz, the elastic modulus decreased from 4300 ± 150 Pa before swelling to 2753 ± 43 Pa after swelling equilibrium. This decrease can be mainly attributed to water uptake and hydration-induced expansion of the polymer network, which reduced the effective load-bearing density of the swollen microgels. A similar concentration-dependent decrease caused by surfactant addition was still observed after swelling. At 0.1 Hz, the elastic modulus decreased from 2753 ± 43 Pa for the swollen SMG system to 1950 ± 51 Pa for the swollen S/SMG system containing 0.5 wt% surfactant.

The modulus reduction after surfactant uptake should not be interpreted simply as a consequence of reduced polymer-network density. Although the smaller swollen size of S/SMG may correspond to a slightly higher nominal polymer-network density, the measured elastic modulus reflects the apparent mechanical response of the packed swollen microgel system rather than only the intrinsic density of an individual microgel particle. Surfactant association with the hydrated microgel network may weaken polymer-chain interactions, modify the interparticle contact structure, increase local chain mobility, and introduce a more hydrated or lubricated interfacial environment. These effects can reduce the effective load-bearing contacts among swollen particles and thereby lead to a lower apparent elastic modulus. The concentration at which the modulus reduction tended to level off shifted slightly to higher surfactant concentrations after swelling, which may be related to the larger accessible surface/network region of the swollen microgels.

Overall, surfactant uptake and association moderately reduced the apparent elastic modulus without changing the basic frequency-dependent elastic-response pattern of the microgels. This reduction in modulus may facilitate particle deformation during pore-throat passage while preserving the elastic recovery required for flow-resistance development in porous media.

#### 2.2.3. Enhanced Electrostatic Stabilization During Storage

**Figure 7:**
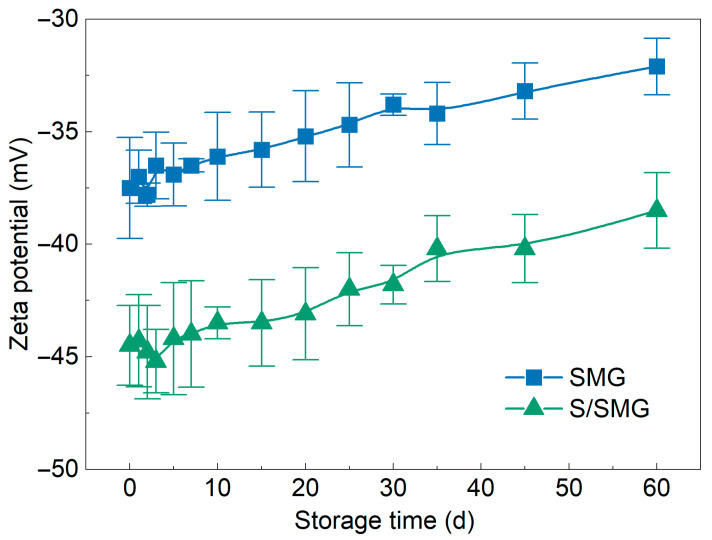
Zeta potential of the SMG and S/SMG systems (*C*_SMG_ = 0.3 wt%, *C*_S_ = 0.2 wt%) as a function of storage time. Symbols and error bars represent mean ± SD (*n* = 3).

Zeta-potential measurements were used to evaluate the effect of surfactant addition on the electrostatic stabilization of the microgel systems during long-term storage. Figure 7 shows the zeta-potential evolution of the SMG system and the S/SMG system (*C*_SMG_ = 0.3 wt%, *C*_S_ = 0.2 wt%) during storage for 60 d.

As shown in Figure 7, the S/SMG system maintained a larger magnitude of negative zeta potential than the SMG system throughout the 60 d storage period. At 0 d, the zeta potential changed from −37.5 ± 2.25 mV for the SMG system to −44.5 ± 1.77 mV for the S/SMG system, indicating that surfactant addition made the apparent zeta potential more negative. After 60 d, the magnitude of the negative zeta potential decreased slightly in both systems, with the SMG system changing from −37.5 ± 2.25 to −32.1 ± 1.26 mV and the S/SMG system changing from −44.5 ± 1.77 to −38.5 ± 1.68 mV. Nevertheless, the S/SMG system still maintained a more negative zeta potential than the SMG system after long-term storage. These results indicate that surfactant addition enhanced electrostatic stabilization and helped the S/SMG system retain stronger interparticle electrostatic repulsion during storage.

The more negative zeta potential of the S/SMG system may be related to the association of the hydroxysulfobetaine surfactant with SMGs, as supported by the UPLC–ELSD, FTIR, and XPS evidence in Section 2.1.3. Although hydroxysulfobetaine is zwitterionic, its sulfonate-containing head group and associated hydration layer may be located near the slipping plane after surfactant association with SMGs. In addition, surfactant association may modify the exposure and distribution of ionizable groups in the sulfonated polyacrylamide microgel network. These effects can lead to a larger apparent negative zeta potential.

The time-dependent decrease in the magnitude of the negative zeta potential during storage may be associated with gradual hydration and structural relaxation of the microgels, ion exchange with the brine, counterion screening, and rearrangement or compression of the electrical double layer during long-term storage at 45 °C. Because both the SMG and S/SMG systems were stored in the same brine environment, this time-dependent change occurred in both systems. However, the S/SMG system remained more negatively charged throughout storage, suggesting that surfactant uptake and association helped maintain stronger electrostatic stabilization.

From the perspective of transport in porous media, stronger electrostatic stabilization is beneficial because it helps maintain particle discreteness before injection and may reduce the risk of nonspecific aggregation during migration. This behavior is favorable for preserving transportability and delaying premature accumulation in the near-wellbore region.

#### 2.2.4. Improved Shear Resistance

**Figure 8:**
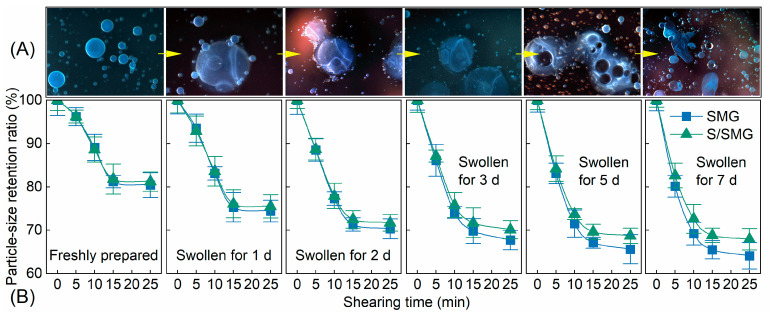
(**A**) Representative microscopic images and (**B**) particle size retention ratio as a function of shearing time for the SMG and S/SMG systems at different swelling states, including freshly prepared samples and samples swollen for 1, 2, 3, 5, and 7 d. Arrows in (**A**) indicate the sequence of representative microscopic images with increasing swelling time. Symbols and error bars in (**B**) represent mean ± SD (*n* = 3).

Shear resistance is important for the field application of soft microgels because the particles are exposed to repeated shear during surface preparation, injection, and transport through pore-throat networks. To evaluate the structural integrity of the SMG and S/SMG systems under shear, the particle size retention ratio was monitored as a function of shearing time at different swelling states. The results are shown in Figure 8 ; Figure 8A presents representative microscopic images and Figure 8B quantifies particle size retention.

As shown in Figure 8B, both systems exhibited a rapid decrease in particle size retention during the initial shearing period, followed by a gradual approach to a quasi-steady level. This two-stage behavior indicates that a fraction of shear-sensitive structures was disrupted at early times, whereas the remaining particles became relatively stable under the imposed shear condition.

Surfactant addition generally improved shear resistance across different swelling states, as reflected by the higher mean particle size retention ratio of the S/SMG system compared with the SMG system at the same swelling time and shearing duration. This difference became more evident after swelling, when the enlarged particles were more susceptible to shear-induced deformation and breakup.

The improved shear resistance is consistent with the surfactant uptake and association behavior established in Section 2.1 and with the enhanced electrostatic stabilization discussed in Section 2.2.3. Surfactant association may reduce direct particle contact, modify particle–particle interactions, and suppress the formation of unstable aggregates, thereby lowering the probability of shear-induced breakup. From an engineering perspective, improved shear resistance helps preserve particle integrity during transport and increases the fraction of effective particles that can reach deeper regions of porous media.

### 2.3. From Surfactant Uptake and Association to Transport Behavior in Porous Media

**Figure 9:**
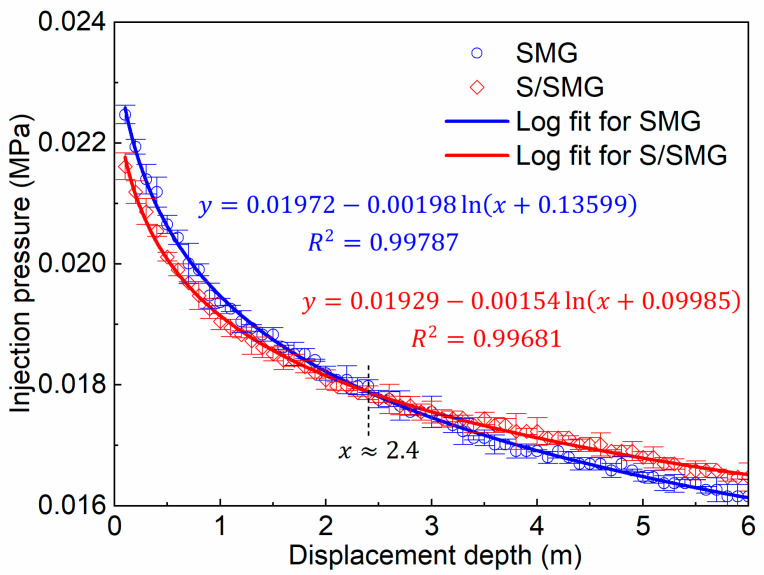
Injection pressure as a function of displacement depth for the SMG and S/SMG systems in serial core-flooding experiments. Symbols and error bars represent mean ± SD (*n* = 3), and solid lines represent logarithmic fits based on the mean values.

To evaluate the deep transport and flow-resistance behavior of the SMG and S/SMG systems in porous media, serial core-flooding experiments consisting of 60 consecutive stages were conducted. This design was intended to simulate the repeated pore-scale shear, retention, and progressive dilution that the tested systems may experience during long-distance migration in reservoirs. For each stage, the stabilized injection pressure was extracted and mapped to the equivalent cumulative displacement depth (*L*), yielding the pressure–distance relationship shown in Figure 9.

As shown in Figure 9, the mean injection pressure generally decreased with increasing displacement depth for both systems. This overall decay reflects the progressive loss of transport effectiveness during migration, which can arise from particle fragmentation, retention within the porous medium, and dilution during continuous flow. However, the two systems showed different pressure-decay behaviors, and a crossover was observed at approximately *L* ≈ 2.4 m.

For *L* < 2.4 m, the SMG system showed a slightly higher mean injection pressure than the S/SMG system. This result is consistent with the somewhat larger swollen size and higher elastic response of the SMG system in the near-inlet region (Section 2.2.1 and Section 2.2.2), which may favor earlier local restriction and flow resistance. Beyond approximately *L* = 2.4 m, the trend reversed. The mean injection pressure of the S/SMG system became slightly higher than that of the SMG system, and this difference was maintained during further transport. At *L* = 6.0 m, the remaining injection pressure of the S/SMG system was 0.01648 ± 0.00023 MPa, which was slightly higher than that of the SMG system (0.01616 ± 0.00028 MPa). Although the absolute difference at the far end was modest and the error bars partially overlapped at some transport distances, the repeated experiments showed the same crossover tendency and a slower pressure-decay trend for the S/SMG system. Therefore, this result should be interpreted as a consistent tendency toward improved deep-transport behavior rather than a large pressure enhancement.

This crossover behavior is consistent with the surfactant-uptake-induced changes in microgel properties described in Section 2.1 and Section 2.2. First, surfactant uptake and association, supported by the UPLC–ELSD, FTIR, and XPS evidence in Section 2.1, limited excessive swelling (Section 2.2.1) and improved shear resistance (Section 2.2.4), allowing a larger fraction of microgels to remain effective during repeated transport. Second, the enhanced electrostatic stabilization during storage (Section 2.2.3) helped reduce the tendency toward nonspecific particle association before and during migration. As a result, although the elastic modulus of individual microgels was moderately reduced (Section 2.2.2), the S/SMG system was more likely to retain effective particles during deeper transport, where their cumulative retention and accumulation sustained higher flow resistance. Therefore, surfactant addition did not simply change the initial flow-resistance response but favored the development of flow resistance in deeper regions of the porous medium.

## 3. Conclusions

1. The hydroxysulfobetaine surfactant was taken up by SMGs through a measurable and saturable association process. The uptake behavior was well described by pseudo-second-order kinetics and a Langmuir-type saturation model, with an experimental equilibrium apparent uptake capacity of 1.868 ± 0.089 mg/g and a theoretical maximum apparent uptake capacity of 3.627 mg/g. FTIR and XPS analyses further provided spectroscopic evidence for surfactant association with SMGs, especially near the particle surface.
2. Surfactant addition mildly reduced the swollen size and swelling ratio of the microgels while preserving substantial swelling ability. Compared with the SMG system, the swollen median size (*D*_50_) at 15 d decreased from 15.72 ± 1.49 to 14.90 ± 1.37 μm, and the corresponding swelling ratio decreased slightly from 7.09 to 6.45. This indicates that surfactant uptake and association moderately limited microgel swelling while maintaining substantial swelling ability under the tested conditions.
3. Surfactant uptake and association moderately reduced the elastic modulus of the microgels without changing their basic frequency-dependent elastic-response pattern. This change may facilitate particle deformation during pore-throat passage while preserving the elastic response required for flow-resistance development in porous media.
4. The S/SMG system showed stronger electrostatic stabilization and better shear resistance than the SMG system. The zeta potential changed from −44.5 ± 1.77 mV at 0 d to −38.5 ± 1.68 mV after 60 d for the S/SMG system, compared with −37.5 ± 2.25 to −32.1 ± 1.26 mV for the SMG system. In addition, particle size retention during shearing was generally 0.8–3.8 percentage points higher for the S/SMG system over 0–7 d of swelling, indicating improved structural stability during repeated shear exposure.
5. The coupled microscale changes improved the deep transport behavior of the microgels in porous media. In serial core-flooding experiments, a crossover in injection-pressure response was observed at approximately *L* ≈ 2.4 m. The SMG system showed slightly higher flow resistance at shorter distances, whereas the S/SMG system maintained a slightly higher pressure response beyond this point. At *L* = 6.0 m, the injection pressure of the S/SMG system was 0.01648 ± 0.00023 MPa, slightly higher than that of the SMG system (0.01616 ± 0.00028 MPa). These results indicate that surfactant uptake and association help preserve the transport effectiveness of SMGs during long-distance migration and improve the deep profile-control performance of the system.

## 4. Materials and Methods

### 4.1. Materials

**Figure 10:**
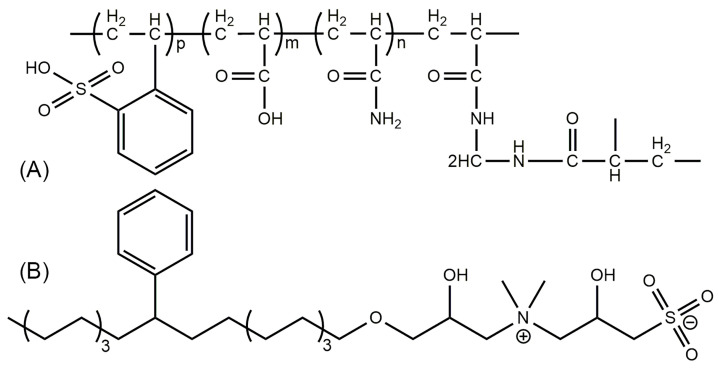
Molecular structures of (**A**) the sulfonated polyacrylamide-based polymer material used for the soft microgels (SMGs) and (**B**) the hydroxysulfobetaine surfactant used in this study. The + and − signs in (**B**) denote the quaternary ammonium cation and sulfonate anion, respectively. Adapted from ref. [35] under the terms of the Creative Commons Attribution 4.0 International License CC BY 4.0).

Commercial polymer microspheres supplied by the Research Institute of Petroleum Exploration and Development, PetroChina, Beijing, China, were used in this study. According to the molecular structure provided for this material (Figure 10A [35]), these particles are sulfonated polyacrylamide-based, preformed, crosslinked, and swellable polymer particles; therefore, they are referred to hereafter as soft microgels (SMGs). The hydroxysulfobetaine surfactant used in this study was prepared in our laboratory, and its molecular structure is shown in Figure 10B [35,36].

Formation water and injection water from the Daqing Oilfield, Daqing, China, were used in this study, and their ionic compositions are listed in Table 2. Before use, both brines were filtered through a 0.22 μm membrane and degassed.

**Figure 11:**
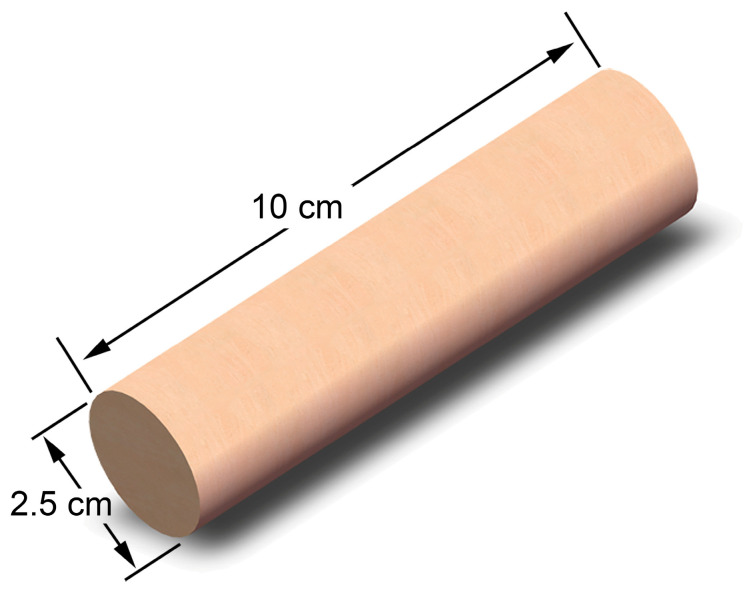
Schematic illustration of the appearance and structure of the artificial core.

Artificial homogeneous cylindrical cores (2.5 cm in diameter and 10 cm in length; Figure 11) were prepared from epoxy-resin-consolidated quartz sand. The water permeability was 814.9 × 10^−3^ μm^2^ (approximately 814.9 mD).

### 4.2. Preparation of SMG and S/SMG Systems

All systems were prepared with injection water (Table 1) as the continuous phase at 25 ± 1 °C under mechanical stirring. The required amounts of SMGs and, when applicable, surfactant were gradually added to the aqueous phase and then stirred for 30 min to obtain a homogeneous dispersion.

For the SMG system, the SMG concentration was fixed at 0.3 wt%. For the surfactant/soft microgel composite systems, hereafter denoted as S/SMG systems, the SMG concentration was also fixed at 0.3 wt%, while the surfactant concentration (*C*_S_) ranged from 0.1 to 0.6 wt% at intervals of 0.1 wt%.

### 4.3. Characterization of Surfactant Uptake and Association with SMGs

Batch uptake experiments were conducted to quantify the apparent uptake of the hydroxysulfobetaine surfactant by soft microgels (SMGs). The residual surfactant concentration in the aqueous phase was determined by ultra-performance liquid chromatography with evaporative light-scattering detection (UPLC–ELSD). A series of surfactant standard solutions with different concentrations were prepared in a 1:1 (*v*/*v*) mixture of injection water and methanol. The standards were stored at 4 °C for 12 h and filtered through a 0.22 μm membrane before analysis. Chromatographic separation was carried out on a Waters UPLC system (Waters, Milford, MI, USA) equipped with a BEH C18 column, and surfactant concentration was quantified using an ELSD calibration curve.

Methanol was used only for standard preparation and, when necessary, sample pretreatment for UPLC–ELSD analysis. All surfactant uptake experiments were conducted in injection water without alcohol.

#### 4.3.1. Surfactant Uptake Kinetics

SMGs (0.30 g) were added to 100 mL of surfactant solution prepared in injection water at an initial concentration of 2000 mg/L. The mixture was shaken in a thermostatic water bath at 45 °C and 150 rpm. At predetermined time intervals, aliquots were withdrawn, centrifuged at 12,000 rpm (approximately 13,500× *g*) for 10 min, and filtered through a 0.22 μm membrane. The residual surfactant concentration in the supernatant (*C*_t_) was then determined by UPLC–ELSD. The apparent surfactant uptake amount at time *t*, *Q*_t_ (mg/g), was calculated using Equation (4):

(4) $$Q_{t}=\frac{\left(C_{0}-C_{t}\right)V}{m},$$

where *C*_0_ and *C*_t_ (mg/L) are the initial and residual surfactant concentrations, respectively; *V* (L) is the solution volume; and *m* (g) is the mass of SMGs.

#### 4.3.2. Uptake Isotherms

Uptake isotherms were determined by adding SMGs (0.30 g) to 100 mL of surfactant solutions prepared in injection water with initial concentrations ranging from 50 to 5000 mg/L. The mixtures were agitated at 45 °C until the uptake equilibrium was reached. The samples were then centrifuged and filtered as described above, and the equilibrium surfactant concentration (*C*_e_) was measured by UPLC–ELSD. The apparent equilibrium uptake capacity, *Q*_e_, was calculated using Equation (4) with Ct replaced by *C*_e_.

#### 4.3.3. FTIR and XPS Characterization of Surfactant Association with SMGs

To provide spectroscopic evidence for surfactant association with SMGs, Fourier-transform infrared spectroscopy (FTIR) and X-ray photoelectron spectroscopy (XPS) were performed on pristine SMGs, the pure hydroxysulfobetaine surfactant, and washed S/SMG. The surfactant-associated SMG sample was prepared by adding SMGs to the hydroxysulfobetaine surfactant solution in injection water and aging the suspension at 45 °C until uptake equilibrium was reached. The solid SMGs were then separated by centrifugation and gently washed to remove unbound surfactant. The washing step was repeated until the surfactant concentration in the supernatant became negligible, as confirmed by UPLC–ELSD. The collected samples were freeze-dried before spectroscopic analysis.

FTIR spectra were recorded using a Nicolet Magna-IR 750 FTIR spectrometer (Nicolet Instrument Corporation, Madison, WI, USA) in the range of 4000–500 cm^−1^ with a spectral resolution of 4 cm^−1^. The spectra of pristine SMGs, the pure hydroxysulfobetaine surfactant, and washed S/SMG were compared to identify surfactant-related functional groups remaining associated with SMGs after uptake and washing.

XPS analysis was conducted using an X-ray photoelectron spectrometer (XPS/ISS/UPS, SPECS Surface Nano Analysis GmbH, Berlin, Germany). Survey spectra were collected to determine the surface elemental composition of pristine SMGs and washed S/SMG, and high-resolution C 1s and N 1s spectra were collected to analyze the surface chemical-state changes after surfactant association. The binding energies were calibrated using the C 1s peak at 284.8 eV. Peak fitting was performed to evaluate changes in the surface chemical environment after surfactant association.

### 4.4. Physicochemical Characterization of Soft Microgels

#### 4.4.1. Morphology and Particle Size

The morphology of the soft microgels was examined by scanning electron microscopy (S-3400N, Hitachi, Tokyo, Japan), and representative SEM images are presented. Particle size distributions were measured at 25 °C using a laser particle size analyzer (LA-300, Horiba, Kyoto, Japan).

#### 4.4.2. Swelling Behavior

Samples were swollen at 45 °C for predetermined swelling times. After swelling, particle size distributions were measured using the laser particle size analyzer described above. The swelling ratio was defined as the ratio of the median particle size at swelling time *t* (*D*_t_) to the initial median particle size (*D*_0_).

#### 4.4.3. Frequency-Dependent Elastic Response

Excess free water in the SMG and S/SMG systems was removed with filter paper to obtain packed swollen microgels. Frequency sweep tests were then performed at 45 °C using a rheometer (MCR 302, Anton Paar, Graz, Austria) over an oscillation frequency, *f*, range of 0.1–100 Hz. A fixed strain within the linear response region was applied, and the elastic modulus was used as a deformation-related parameter to characterize the frequency-dependent elastic response of the packed swollen microgels.

#### 4.4.4. Zeta-Potential Measurement and Electrostatic Stability Evaluation

The zeta potentials of the SMG system (0.3 wt%) and a representative S/SMG system (0.3 wt% SMGs + 0.2 wt% surfactant) were measured at 25 °C using a Zetasizer Nano ZS (Malvern Instruments Ltd., Malvern, UK). To evaluate electrostatic stability during long-term storage, the samples were stored at 45 °C, and the zeta potential was measured periodically.

#### 4.4.5. Shear Resistance Evaluation

Mechanical shearing was applied to the SMG and S/SMG systems using a high-shear disperser (FLUKO FM200A, FLUKO, Shanghai, China). Samples were sheared for 1, 5, and 10 min. The median particle size (*D*_50_) was measured before and after shearing using the laser particle size analyzer described above. The particle size retention ratio, *R*_D_, was calculated using Equation (5):

(5) $$R_{D}=\frac{D_{s}}{D_{0}}\times 100\%,$$

where *D*_0_ and *D*_S_ are the median particle sizes (*D*_50_) before and after shearing, respectively. The macroscopic morphology of the sheared microgels was also observed using a stereomicroscope (SteREO Discovery.V12, Carl Zeiss, Jena, Germany), and representative microscopic images are presented.

### 4.5. Serial Core Flooding and Long-Distance Transport Evaluation

**Figure 12:**
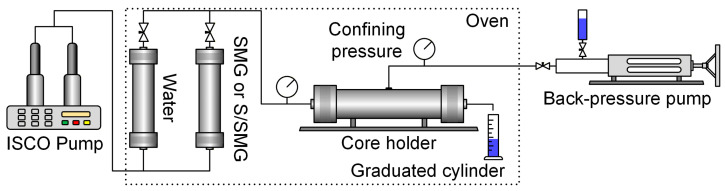
Schematic of the serial core-flooding setup and stage-to-stage transfer procedure used to simulate long-distance transport in porous media.

A serial core-flooding protocol was used to simulate the long-distance transport of chemical agents in porous media and to evaluate the evolution of injection pressure along the transport path. The experimental setup is shown in Figure 12. Artificial homogeneous cylindrical cores (2.5 cm in diameter and 10 cm in length) were mounted in a core holder placed in a temperature-controlled oven at 45 °C. A confining pressure was applied, and the outlet back pressure was maintained at 2.0 MPa during flooding. The injection flow rate was fixed at 0.3 mL/min.

Before flooding, each core was vacuum-evacuated, saturated with formation water, and water-flooded to determine its initial permeability. The serial flooding experiment consisted of 60 stages (cores 1–60). In stage 1, core 1 was injected with 10 PV of the tested system (SMG or S/SMG), and the injection pressure was recorded continuously. The effluent from core 1 was collected in a graduated cylinder and used as the injected fluid for stage 2. In stage 2, core 2 was injected with 10 PV of the effluent from stage 1 under the same flow rate and temperature conditions. This stage-to-stage transfer procedure was repeated sequentially to core 60. An injection volume of 10 PV per stage was selected to ensure sufficient effluent volume for transfer while minimizing liquid loss.

For each stage, the injection pressure reached a quasi-steady value after approximately 4–5 PV of injection. A representative steady-state pressure was therefore extracted for subsequent analysis. The transport distance was defined as the cumulative core length traversed by the chemical agent in the serial sequence:

(6) L=N×10 cm,

for stage *N*, where *L* is the cumulative transport distance at the end of stage *N*. The steady-state pressure obtained in stage *N* therefore represents the pressure response over the transport segment from (*N* − 1) × 10 to *N* × 10 cm. By compiling one steady-state pressure value from each stage, the relationship between injection pressure and transport distance was constructed and then fitted to quantify pressure evolution along the transport path. The full pressure–PV profiles for all 60 stages are not presented in the main text.

The SMG and S/SMG systems were each tested in three independent serial core-flooding runs under the same experimental protocol. The pressure values at the same cumulative displacement depth were averaged for subsequent analysis.

### 4.6. Replicates and Data Analysis

Unless otherwise stated, all quantitative measurements were performed in three independent experiments. The results are reported as mean ± standard deviation (SD), and the error bars in the figures represent SD. When the error bars are smaller than the symbols, they may not be clearly visible.

For surfactant uptake kinetics and uptake isotherms, the apparent uptake capacity at each contact time or equilibrium concentration was calculated separately from three independent uptake experiments, and the mean apparent uptake capacities were used for kinetic and isotherm model fitting. For particle size analysis and swelling experiments, *D*_50_ values were obtained from three independent measurements and reported as mean ± SD. For rheological measurements, the elastic modulus at each frequency was averaged from three independent measurements. For zeta-potential measurements, each reported value represents the mean ± SD from three independent measurements. For shear-resistance evaluation, the particle size retention ratio was calculated from *D*_50_ values measured before and after shearing in three independent experiments.

For serial core-flooding experiments, the SMG and S/SMG systems were each tested in three independent serial flooding runs under the same core dimensions, permeability range, injection rate, temperature, confining pressure, and back-pressure conditions. For each stage, the steady-state injection pressure was extracted from the quasi-steady pressure region after approximately 4–5 PV of injection. The pressure values at the same cumulative displacement depth were averaged, and the pressure–distance relationship was constructed using the mean values. The logarithmic fitting was performed using the mean pressure values.

## Figures and Tables

**Table 1:** Comparison of kinetic-model fitting parameters and experimental apparent uptake capacity for hydroxysulfobetaine surfactant uptake by soft microgels (SMGs).

Model	*Q*_e,calc_ (mg/g) ^1^	Rate Constant	*R* ^2^	*Q*_e,exp_ (mg/g) ^2^
Pseudo-first-order ^4^	1.657	*k*_1_ ^3^ = 2.244 day^−1^	0.8929	≈1.868 ± 0.089
Pseudo-second-order ^4^	1.803	*k*_2_ ^3^ = 1.693 g∙mg^−1^∙day^−1^	0.9650	≈1.868 ± 0.089

^1^ *Q*_e,calc_ is the apparent equilibrium uptake capacity calculated from kinetic-model fitting; ^2^ *Q*_e,exp_ is the experimentally measured apparent equilibrium uptake capacity at dynamic equilibrium, expressed as mean ± SD from three independent measurements; ^3^ Time *t* is expressed in days; therefore, the units of *k*_1_ and *k*_2_ are day^−1^ and g·mg^−1^·day^−1^, respectively; ^4^ The kinetic fitting was performed using the mean *Q*_t_ values obtained from three independent uptake measurements.

**Table 2:** Ionic composition of the formation water and injection water used in this study (mg/L).

Water Type	Na^+^	Ca^2+^	Mg^2+^	HCO_3_^−^	Cl^−^	SO_4_^2−^	CO_3_^2−^	Total Salinity
Formation water	2428.00	14.90	7.48	2160.08	2266.88	54.10	197.66	7156.5
Injection water	1265.00	32.10	7.30	1708.56	780.12	9.61	210.07	4012.7

## Data Availability

The data presented in this study are available on request from the corresponding author.

## Abbreviations

The following abbreviations are used in this manuscript:

SMG	Sulfonated polyacrylamide soft microgel
S/SMG	Hydroxysulfobetaine surfactant/soft microgel composite system
EOR	Enhanced oil recovery
UPLC–ELSD	Ultra-performance liquid chromatography with evaporative light-scattering detection
FTIR	Fourier-transform infrared spectroscopy
XPS	X-ray photoelectron spectroscopy
*D*_50_	Median particle size
*C*_S_	Surfactant concentration
*C*_SMG_	Soft microgel concentration
*Q*_t_	Apparent surfactant uptake amount at time t
*Q*_e_	Apparent equilibrium surfactant uptake capacity
*Q*_e,calc_	Calculated apparent equilibrium uptake capacity
*Q*_e,exp_	Experimental apparent equilibrium uptake capacity
*Q*_m_	Theoretical maximum apparent uptake capacity
*C*_e_	Equilibrium surfactant concentration in the aqueous phase
*C*_0_	Initial surfactant concentration
*C*_t_	Residual surfactant concentration at time t
*R*_D_	Particle size retention ratio
